# A real-world pharmacovigilance study of bempedoic acid plus ezetimibe fixed-dose combination based on the FDA FAERS database

**DOI:** 10.1097/MD.0000000000047898

**Published:** 2026-02-28

**Authors:** Bing Zhu, Yitong Ma, Qiqi Shao, Jun Cui, Zhenyan Fu

**Affiliations:** a The First Affiliated Hospital of Xinjiang Medical University, Urumqi, Xinjiang, China.

**Keywords:** adverse drug events, BCPNN, bempedoic acid plus ezetimibe fixed-dose combination, FAERS, MGPS, PRR, ROR

## Abstract

The bempedoic acid plus ezetimibe fixed-dose combination (BAPEFC) is a novel lipid-lowering agent that was successively marketed in regions such as the United States and the European Union in 2020. It significantly reduces low-density lipoprotein cholesterol through a dual mechanism of inhibiting adenosine triphosphate-citrate lyase and Niemann-Pick C1-like 1. Although clinical trials have confirmed its efficacy, its long-term safety in the real world requires further evaluation. All adverse event reports related to BAPEFC from the first quarter of 2020 to the first quarter of 2025 were extracted from the FDA Adverse Event Reporting System (FAERS) database. After data standardization, a comprehensive evaluation of adverse reaction signals was performed using the reporting odds ratio (ROR), proportional reporting ratio, Bayesian confidence propagation neural network, and multi-item gamma Poisson shrinker methods. Subgroup analyses by sex and age were further conducted for newly identified adverse drug reactions. A total of 875 reports involving at least 1 BAPEFC agent were identified in FAERS. The preferred term with the highest number of reports was arthralgia (n = 103), while the strongest positive signal was for tendon discomfort (n = 3, ROR = 135.27, proportional reporting ratio = 135.03, information component = 7.06, empirical Bayes geometric mean = 133.36). At the system organ class level, musculoskeletal and connective tissue disorders had the highest ROR value (ROR = 6.02, 95% confidence interval [CI]: 5.46–6.63). The newly identified signal for “Blood Triglycerides Increased” showed statistically significant ROR signals in both male and female subgroups (ROR = 36.59 [95% CI: 15.16–88.32] vs ROR = 34.78 [95% CI: 15.57–77.67]), and the adjusted Bonferroni-*P* values were statistically significant (Bonferroni-*P* < .0001). Subgroup analysis of the combined data for the newly identified “Pancreatitis” and “Pancreatitis Acute” showed statistically significant ROR signals in both the non-elderly subgroup (<65 years) and the elderly subgroup (≥65 years) (ROR = 10.43 [95% CI: 4.32–25.18] vs ROR = 5.88 [95% CI: 2.2–15.71]), and the adjusted Bonferroni-*P* values were statistically significant (Bonferroni-*P* = .0003 vs Bonferroni-*P* = .0106). Based on the FAERS database, this study provides the first comprehensive and systematic analysis of adverse drug events associated with BAPEFC. It newly identifies several adverse drug reactions not mentioned in the prescribing information, such as blood triglycerides increased, pancreatitis, and pancreatitis acute, thereby offering evidence for clinical monitoring and identification of potential risks associated with BAPEFC.

## 1. Introduction

Hypercholesterolemia is a significant risk factor for cardiovascular diseases. Elevated low-density lipoprotein cholesterol (LDL-C) poses a major global public health threat, contributing to millions of cardiovascular deaths annually.^[1]^ Although statins remain the cornerstone of lipid-lowering therapy, some patients are unable to tolerate full treatment due to adverse effects such as myalgia, and monotherapy often fails to achieve LDL-C targets. Ballantyne et al^[2]^ found that among statin-intolerant patients with hypercholesterolemia already on ezetimibe, the addition of bempedoic acid resulted in a further 28.5% reduction in LDL-C compared with the addition of placebo (*P* < .001, with LDL-C decreasing by 23.5% in the bempedoic acid group and increasing by 5.0% in the placebo group).

Bempedoic acid is a prodrug that reduces hepatic cholesterol synthesis by being metabolized to its coenzyme A form in the liver. This active metabolite inhibits adenosine triphosphate-citrate lyase, an enzyme upstream of 3-hydroxy-3-methylglutaryl-coenzyme A reductase in the cholesterol biosynthesis pathway,^[3]^ thereby lowering the incidence of cardiovascular events.^[4]^ Wang et al^[5]^ conducted a retrospective data mining study of real-world adverse events associated with bempedoic acid and found that the most frequently involved system organ classes (SOCs) were musculoskeletal and connective tissue disorders and hepatobiliary disorders. They also identified adverse events with signals of higher strength, such as low-density lipoprotein abnormality, elevated blood uric acid, and biliary colic. Furthermore, new adverse reaction signals, including esophageal spasm, angina, and apathy, were reported. Ezetimibe reduces cholesterol absorption by binding to and inhibiting the Niemann-Pick C1-like 1 transporter. Han et al^[6]^ analyzed adverse events associated with ezetimibe using the FDA Adverse Event Reporting System (FAERS) database from 2004 to 2023. They found that the most common SOCs for ezetimibe were musculoskeletal and connective tissue disorders, followed by gastrointestinal disorders. The most frequent positive signals of adverse reactions were myalgia (n = 1620, reporting odds ratio [ROR] = 18.69), arthralgia (n = 534, ROR = 2.48), and pain in extremity (n = 499, ROR = 3.07). Furthermore, new adverse reactions for ezetimibe were identified, including unstable angina, crush syndrome, autoscopy, and hearing impairment.^[7]^

The bempedoic acid plus ezetimibe fixed-dose combination (BAPEFC), as a novel lipid-lowering agent, has been approved in regions such as the United States and the European Union since 2020. Clinical studies on BAPEFC have demonstrated that this dual-mechanism approach produces a synergistic lipid-lowering effect, reducing LDL-C by 36.2%, which is significantly superior to that of monotherapy (ezetimibe: 23.2%; bempedoic acid: 17.2%) and placebo (+1.8%).^[8]^ Furthermore, it can further reduce the risk of cardiovascular events.^[9–12]^ Although clinical trials have confirmed its efficacy, the post-marketing safety profile of BAPEFC still requires long-term monitoring, necessitating the use of the FAERS database to identify new risk signals associated with this fixed-dose combination.

## 2. Methods

### 2.1. Data source and processing

The FAERS database serves as a key source for post-marketing safety surveillance of all approved drugs in the United States, employing the Medical Dictionary for Regulatory Activities preferred term (PT) to define adverse drug events (ADEs, https://www.meddra.org/). Based on the market approval timeline of BAPEFC, this study extracted FAERS reports from the first quarter of 2020 to the first quarter of 2025. The original datasets were obtained from the official Food and Drug Administration (FDA) website (https://fis.fda.gov/extensions/FPD-QDE-FAERS/FPD-QDE-FAERS.html). All relevant reports were first screened by searching for the drug’s trade names and generic names (NEXLIZET, NUSTENDI, BEMPEDOIC ACID EZETIMIBE, EZETIMIBE BEMPEDOIC ACID, BEMPEDOIC ACID AND EZETIMIBE SPECIFIC SUBSTANCE SUB8456, BEMPEDOIC ACID AND EZETIMIBE SPECIFIC SUBSTANCE SUB12581). Only reports in which the drug was designated as the primary suspect were retained. The FAERS data related to BAPEFC consist of 7 data files: demographic (DEMO), drug (DRUG), reaction (REAC), outcome, report sources, therapy, and indication. To address the common issue of duplicate cases in spontaneous reporting systems, we followed the FDA-recommended deduplication procedures. Using the DEMO table, we sorted reports by primary identifier (PRIMARYID), case identifiers (CASEID), and FDA receipt date (FDA_DT). For reports sharing the same CASEID, the entry with the most recent FDA_DT was kept. If both CASEID and FDA_DT were identical, the record with the largest PRIMARYID was retained. General information on ADEs associated with BAPEFC was collected, including age, sex, reporting year, reporting region, reporter type, and clinical outcomes.

### 2.2. Signal screening

Four disproportionality analysis methods were employed for comprehensive signal detection: the ROR, the proportional reporting ratio (PRR), the Bayesian confidence propagation neural network (BCPNN), and the empirical Bayes geometric mean (EBGM). ROR and PRR are straightforward and intuitive but can be unstable with small sample sizes; PRR generally provides higher specificity than ROR. BCPNN and EBGM incorporate prior distributions and shrinkage effects, which significantly reduce false-positive signals arising from very low report counts and thus enhance the reliability of signal detection. The information component (IC), derived from BCPNN theory, compares observed versus expected frequencies of drug–ADE associations and integrates known probability differences in the background data to develop a sensitive indicator for identifying new disproportionality signals. Notably, EBGM is the core output of the multi-item gamma Poisson shrinker (MGPS) algorithm. By integrating ROR, PRR, BCPNN, and EBGM, this study leveraged the strengths of different detection methods to comprehensively identify safety signals.

### 2.3. Data analysis

**Figure 1.**
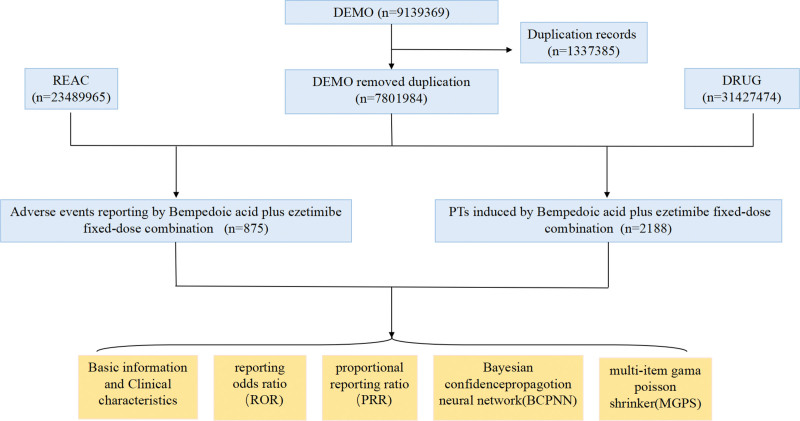
Flowchart of the selection process for adverse drug reports associated with bempedoic acid plus ezetimibe fixed-dose combination (BAPEFC) from the FDA Adverse Event Reporting System (FAERS) database (Q1 2020 to Q1 2025). BCPNN = Bayesian confidence propagation neural network, DEMO = demographic, DRUG = drug, FDA = Food and Drug Administration, MGPS = multi-item gamma Poisson shrinker, PRR = proportional reporting ratio, PTs = preferred terms, REAC = reaction, ROR = reporting odds ratio.

We performed a descriptive analysis of the characteristics of adverse event reports associated with BAPEFC, including sex, age, reporting year, reporting country, and reporter type. Categorical variables were summarized as frequencies and percentages. Disproportionality analysis was conducted based on the 4-fold table of disproportionality measures (Table 1) to detect safety signals, which relies on comparing the frequency of a target drug-event pair with its background frequency. For BAPEFC, we calculated the ROR and its 95% confidence interval (CI), the PRR and its corresponding χ^2^ value, the IC, and the EBGM to identify potential ADE signals. Higher ROR values indicate a greater likelihood of the adverse event. Positive signals were defined using the following thresholds: number of event reports ≥3 and lower limit of the 95% CI > 1 for ROR; PRR > 2.0 and χ^2^ > 4; IC025 > 0; and EBGM05 > 2 (detailed formulas and thresholds are listed in Table 1). To further evaluate the safety of newly identified positive signals, subgroup analyses were performed according to demographic characteristics such as age and sex. Comparisons within subgroups (e.g., female vs male, <65 years vs ≥65 years) were conducted using the chi-square test or Fisher exact test, with *P* values adjusted by the Bonferroni correction (Bonferroni-*P* < .05 was considered statistically significant). All statistical analyses were performed using R software (version 4.5.0; Vienna, Austria). The study flowchart is presented in Figure 1.

**Table 1:** Formulas and signal detection criteria for ROR, PRR, BCPNN, and EBGM.

Algorithms	Equation	Threshold	
ROR	ROR = *ad*/*bc*	lower limit of 95% CI > 1, *a* ≥ 3	
	$95\%\;CI=e^{In(ROR)\pm 1.96{\left(1/a+1/b+1/c+1/d\right)}^{0.5}}$		
PRR	PRR = *a (c* + *d*)/*c*/(*a* + *b*)	PRR > 2, χ^2^ > 4, *a* > 3	
	χ^2^ = ([*ad*−*bc*]^2^)(*a* + *b* + *c* + *d*)/([*a* + *b*][*c* + *d*][*a* + *c*][*b* + *d*])		
BCPNN	IC = log_2_(*a* [*a* + *b* + *c* + *d*]/[*a* + *c*]/[*a* + *b*])	IC025 > 0	
	95% CI = E (IC) ± 2V (IC)^0.5^		
EBGM	EBGM = *a (a* + *b* + *c* + *d*)/(*a* + *c*)/(*a* + *b*)	EBGM05 > 2	
	$EBGM05=e^{In\;(EBGM)-1.64\;{\left(1/a+1/b+1/c+1/d\right)}^{0.5}}$		

Four-fold table of disproportionality measures	Target ADEs	Other ADEs	Total
Target drugs	*a*	*b*	*a* + *b*
Other drugs	*c*	*d*	*c* + *d*
Total	*a* + *c*	*b* + *d*	*a* + *b* + *c* + *d*

ADEs = adverse drug events, BCPNN = Bayesian confidence propagation neural network, CI = confidence interval, EBGM = empirical Bayes geometric mean, EBGM05 = the lower limit of 95% CI, of the EBGM, IC = information component, IC025 = the lower limit of 95% CI, of the IC, PRR = proportional reporting ratio, ROR = reporting odds ratio.

## 3. Results

### 3.1. Basic information of adverse event reports

From the first quarter of 2020 to the first quarter of 2025, a total of 9,139,369 adverse event reports were recorded in the database. Among these, 875 reports were associated with BAPEFC, corresponding to 2188 PTs for adverse events where the combination was listed as the primary suspect (Fig. 1). The number of reported adverse events for BAPEFC exceeded 100 cases annually over the past 5 years. Furthermore, the reporting frequency has shown a gradual increasing trend in the most recent 3 years: 106 reports in 2023, a peak of 243 reports in 2024, and 195 reports in the first quarter of 2025 alone. These findings highlight the importance of enhanced monitoring for adverse events associated with this combination. The proportion of adverse events reported in females (51.0%) exceeded that in males (31.5%). The most affected age group was 65 to 85 years (25.6%). It is noteworthy that 54.1% of reports lacked age information, which limited our ability to analyze adverse reaction incidence across different age groups. The majority of reports originated from the United States (630 cases) and Great Britain (105 cases). Reports were primarily submitted by consumers and physicians. Among the specified serious outcomes, hospitalization was the most frequent (5.3%; Table 2).

**Table 2:** Basic characteristics of ADEs associated with BAPEFC from the FAERS database.

Factors	Number of events (%)
Year	
2020	41 (4.7%)
2021	148 (16.9%)
2022	142 (16.2%)
2023	106 (12.1%)
2024	243 (27.8%)
2025	195 (22.3%)
Sex	
Female	446 (51.0%)
Male	276 (31.5%)
Unknown	153 (17.5%)
Age	
<18 yr	17 (1.9%)
18–64 yr	155 (17.7%)
65–85 yr	224 (25.6%)
>85 yr	6 (0.7%)
Unknown	473 (54.1%)
Reporter type	
Consumer	396 (45.3%)
Health professional	113 (12.9%)
Pharmacist	66 (7.5%)
Physician	300 (34.3%)
Reported countries	
Austria	9 (1.0%)
Belgium	16 (1.8%)
Switzerland	3 (0.3%)
Germany	84 (9.6%)
Spain	10 (1.1%)
Great Britain	105 (12.0%)
Italy	17 (1.9%)
Luxembourg	1 (0.1%)
United States	630 (72.0%)
Outcome	
Death	6 (0.7%)
Disability	20 (2.3%)
Hospitalization	46 (5.3%)
Life-threatening	9 (1.0%)
Other	794 (90.7%)

ADEs = adverse drug events, BAPEFC = bempedoic acid plus ezetimibe fixed-dose combination, FAERS = FDA Adverse Event Reporting System.

### 3.2. Risk signal mining results

**Figure 2.**
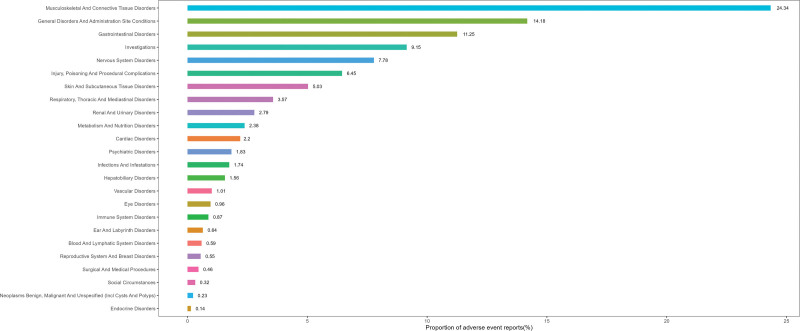
Proportional distribution of adverse drug events (ADEs) associated with BAPEFC at the system organ class (SOC) level in the FAERS database. Percentages represent the proportion of total reports for each SOC. BAPEFC = bempedoic acid plus ezetimibe fixed-dose combination, FAERS = FDA Adverse Event Reporting System.

**Figure 3.**
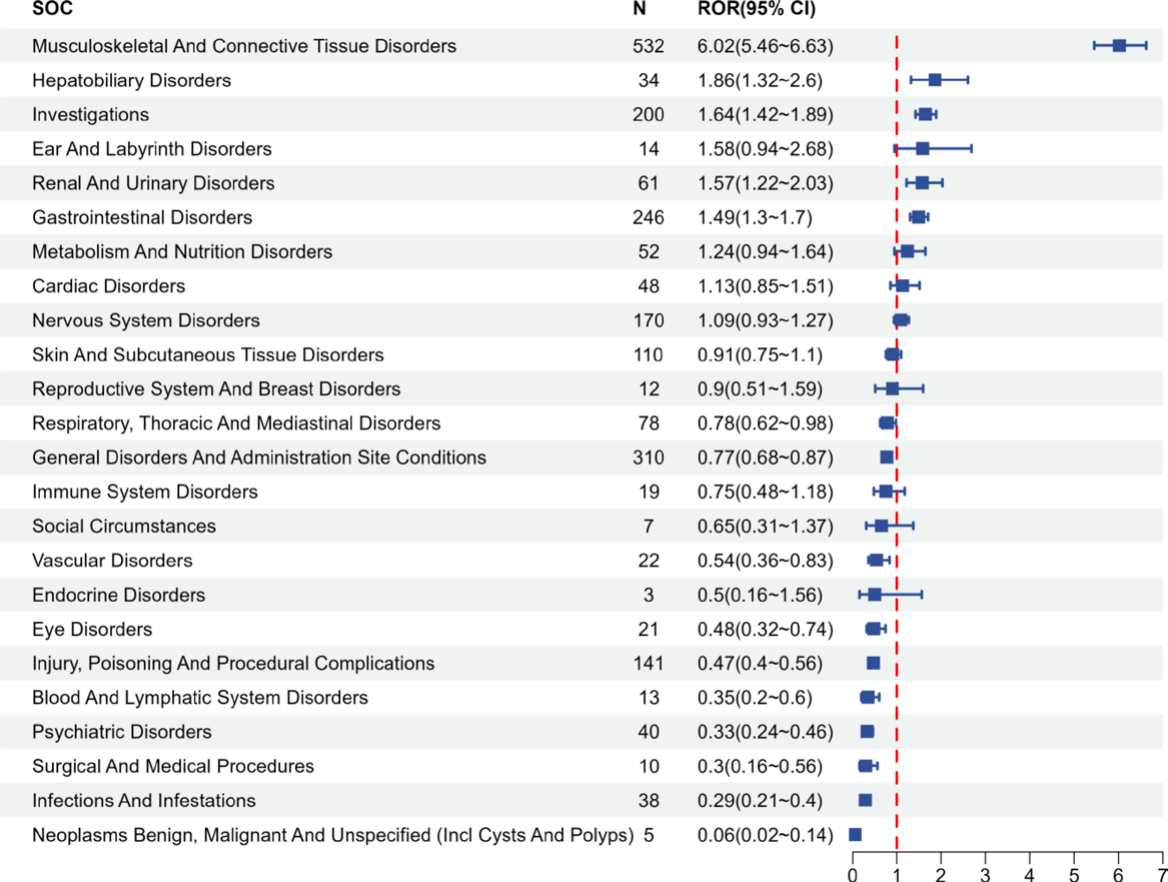
Signal strength of adverse drug events (ADEs) associated with BAPEFC at the system organ class (SOC) level, expressed as reporting odds ratio (ROR) with 95% confidence intervals. Only SOCs with positive signals (lower limit of 95% CI > 1) are labeled. BAPEFC = bempedoic acid plus ezetimibe fixed-dose combination, CI = confidence interval.

The ADEs associated with the BAPEFC involved 24 SOCs, as detailed in Table 3. The results of this study indicated that the adverse events most strongly associated with the use of the BAPEFC primarily occurred in musculoskeletal and connective tissue disorders (24.34%), followed by general disorders and administration site conditions (14.18%), and gastrointestinal disorders (11.25%; Fig. 2). Based on the ROR algorithm, the SOCs identified as positive signals were musculoskeletal and connective tissue disorders (ROR = 6.02, 95% CI: 5.46–6.63), hepatobiliary disorders (ROR = 1.86, 95% CI: 1.32–2.60), investigations (ROR = 1.64, 95% CI: 1.42–1.89), renal and urinary disorders (ROR = 1.57, 95% CI: 1.22–2.03), and gastrointestinal disorders (ROR = 1.49, 95% CI: 1.30–1.70; Fig. 3).

**Table 3:** The signal strength of ADEs associated with BAPEFC at the SOC level in the FAERS database.

System organ class (SOC)	Case reports (n)	ROR (95% CI)	PRR (χ^2^)	EBGM (EBGM05)	IC (IC025)
Musculoskeletal and connective tissue disorders	532	6.02 (5.46–6.63)	4.80 (1683.43)	4.80 (4.35)	2.26 (2.11)
General disorders and administration site conditions	310	0.77 (0.68–0.87)	0.80 (18.53)	0.80 (0.71)	−0.32 (−0.49)
Gastrointestinal disorders	246	1.49 (1.30–1.7 0)	1.43 (34.6)	1.43 (1.25)	0.52 (0.32)
Investigations	200	1.64 (1.42–1.89)	1.58 (45.00)	1.58 (1.36)	0.66 (0.44)
Nervous system disorders	170	1.09 (0.93–1.27)	1.08 (1.11)	1.08 (0.92)	0.11 (−0.12)
Injury, poisoning, and procedural complications	141	0.47 (0.40–0.56)	0.51 (77.09)	0.51 (0.43)	−0.98 (−1.22)
Skin and subcutaneous tissue disorders	110	0.91 (0.75–1.10)	0.91 (0.99)	0.91 (0.75)	−0.13 (−0.41)
Respiratory, thoracic, and mediastinal disorders	78	0.78 (0.62–0.98)	0.79 (4.70)	0.79 (0.63)	−0.35 (−0.67)
Renal and urinary disorders	61	1.57 (1.22–2.03)	1.56 (12.43)	1.56 (1.21)	0.64 (0.25)
Metabolism and nutrition disorders	52	1.24 (0.94–1.64)	1.24 (2.42)	1.24 (0.94)	0.31 (−0.10)
Cardiac disorders	48	1.13 (0.85–1.51)	1.13 (0.74)	1.13 (0.85)	0.18 (−0.24)
Psychiatric disorders	40	0.33 (0.24–0.46)	0.35 (52.43)	0.35 (0.25)	−1.53 (−1.97)
Infections and infestations	38	0.29 (0.21–0.40)	0.30 (63.82)	0.31 (0.22)	−1.71 (−2.15)
Hepatobiliary disorders	34	1.86 (1.32–2.60)	1.84 (13.21)	1.84 (1.31)	0.88 (0.36)
Vascular disorders	22	0.54 (0.36–0.83)	0.55 (8.36)	0.55 (0.36)	−0.87 (−1.45)
Eye disorders	21	0.48 (0.32–0.74)	0.49 (11.40)	0.49 (0.32)	−1.03 (−1.62)
Immune system disorders	19	0.75 (0.48–1.18)	0.75 (1.55)	0.75 (0.48)	−0.41 (−1.04)
Ear and labyrinth disorders	14	1.58 (0.94–2.68)	1.58 (2.98)	1.58 (0.93)	0.66 (−0.14)
Blood and lymphatic system disorders	13	0.35 (0.20–0.60)	0.35 (15.99)	0.35 (0.20)	−1.52 (−2.22)
Reproductive system and breast disorders	12	0.90 (0.51–1.59)	0.90 (0.13)	0.90 (0.51)	−0.15 (−0.94)
Surgical and medical procedures	10	0.30 (0.16–0.56)	0.30 (16.43)	0.30 (0.16)	−1.73 (−2.51)
Social circumstances	7	0.65 (0.31–1.37)	0.66 (1.27)	0.66 (0.31)	−0.61 (−1.57)
Neoplasms benign, malignant, and unspecified (incl cysts and polyps)	5	0.06 (0.02–0.14)	0.06 (75.24)	0.06 (0.03)	−4.04 (−4.97)
Endocrine disorders	3	0.50 (0.16–1.56)	0.50 (1.47)	0.50 (0.16)	−0.99 (−2.24)

ADEs = adverse drug events, BAPEFC = bempedoic acid plus ezetimibe fixed-dose combination, CI = confidence interval, EBGM = empirical Bayes geometric mean, EBGM05 = the lower limit of 95% CI, of the EBGM, FAERS = FDA Adverse Event Reporting System, IC = information component, IC025 = the lower limit of 95% CI, of the IC, PRR = proportional reporting ratio, ROR = reporting odds ratio, SOC = system organ class.

**Figure 4.**
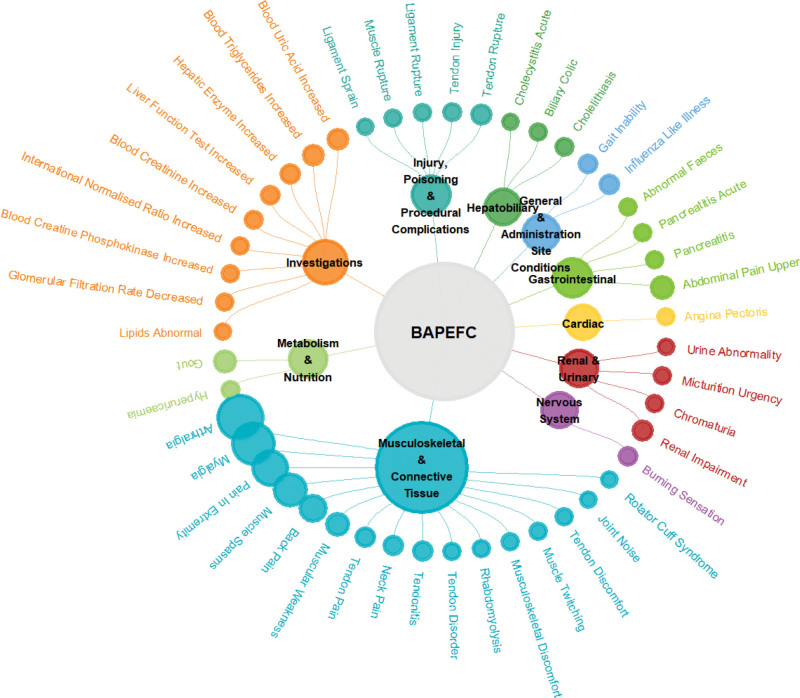
Network plot showing the distribution of ADE signals across system organ classes (SOCs) for BAPEFC. The central node represents the drug and total number of ADE signals; inner ring segments correspond to SOCs, and outer ring segments represent individual preferred terms (PTs) within each SOC. ADE = adverse drug event, BAPEFC = bempedoic acid plus ezetimibe fixed-dose combination.

At the PT level, positive signal detection was performed using 4 distinct algorithms. A total of 47 PTs were concurrently identified as positive signals by all 4 algorithms, spanning 10 SOCs (Table 4 and Fig. 4). Among these, the PTs demonstrating the most prominent signal strengths were as follows: tendon discomfort (n = 4; ROR = 135.27, PRR = 135.03, IC = 7.06, EBGM = 133.36), lipids abnormal (n = 3; ROR = 127.97, PRR = 127.80, IC = 6.98, EBGM = 126.30), tendon injury (n = 8; ROR = 98.28, PRR = 97.92, IC = 6.60, EBGM = 97.05), and blood uric acid increased (n = 17; ROR = 89.36, PRR = 88.67, IC = 6.46, EBGM = 87.96). The PTs with the highest number of reports included arthralgia (n = 103; ROR = 6.91, PRR = 6.64, IC = 2.73, EBGM = 6.63), myalgia (n = 91; ROR = 19.79, PRR = 19.01, IC = 4.25, EBGM = 18.97), pain in extremity (n = 67; ROR = 7.61, PRR = 7.41, IC = 2.89, EBGM = 7.41), and muscle spasms (n = 60; ROR = 11.66, PRR = 11.37, IC = 3.51, EBGM = 11.36). Notably, several PTs not currently mentioned in the prescribing information were identified as positive signals, including blood triglycerides increased (n = 16; ROR = 42.83, PRR = 42.52, IC = 5.40, EBGM = 42.36), pancreatitis (n = 6; ROR = 4.61, PRR = 4.60, IC = 2.20, EBGM = 4.60), and pancreatitis acute (n = 6; ROR = 9.19, PRR = 9.17, IC = 3.20, EBGM = 9.16).

**Table 4:** The positive signal strength of ADEs associated with BAPEFC at the PTs level.

Preferred term (PT)	Case reports	ROR (95% CI)	IC (IC025)	PRR (χ^2^)	EBGM (EBGM05)
Arthralgia	103	6.91 (5.67–8.43)	2.73 (2.36)	6.64 (496.28)	6.63 (5.44)
Myalgia	91	19.79 (16.04–24.41)	4.25 (3.68)	19.01 (1552.96)	18.97 (15.38)
Pain in extremity	67	7.61 (5.97–9.71)	2.89 (2.40)	7.41 (372.83)	7.41 (5.81)
Muscle spasms	60	11.66 (9.02–15.07)	3.51 (2.90)	11.37 (568.14)	11.36 (8.79)
Back pain	39	5.39 (3.93–7.40)	2.41 (1.80)	5.32 (137.05)	5.31 (3.87)
Abdominal pain upper	25	3.84 (2.59–5.70)	1.93 (1.21)	3.81 (51.96)	3.81 (2.57)
Muscular weakness	23	6.93 (4.60–10.46)	2.78 (1.87)	6.87 (115.45)	6.87 (4.55)
Gout	22	38.53 (25.3–58.68)	5.25 (3.26)	38.15 (793.33)	38.02 (24.96)
Blood uric acid increased	17	89.36 (55.34–144.3)	6.46 (3.23)	88.67 (1461.71)	87.96 (54.47)
Blood triglycerides increased	16	42.83 (26.17–70.11)	5.40 (2.92)	42.52 (646.32)	42.36 (25.88)
Tendon rupture	15	52.03 (31.27–86.55)	5.68 (2.90)	51.68 (741.96)	51.43 (30.92)
Renal impairment	14	4.47 (2.65–7.57)	2.15 (1.11)	4.45 (37.51)	4.45 (2.63)
Hepatic enzyme increased	13	5.04 (2.92–8.70)	2.33 (1.19)	5.02 (41.87)	5.02 (2.91)
Tendon pain	13	58.51 (33.87–101.08)	5.85 (2.74)	58.17 (726.59)	57.86 (33.49)
Influenza-like illness	11	5.73 (3.17–10.36)	2.51 (1.20)	5.70 (42.66)	5.70 (3.15)
Neck pain	11	6.08 (3.36–11.00)	2.60 (1.25)	6.06 (46.44)	6.05 (3.35)
Tendonitis	11	26.28 (14.52–47.56)	4.71 (2.24)	26.15 (265.51)	26.09 (14.42)
Gait inability	9	4.89 (2.54–9.41)	2.28 (0.90)	4.87 (27.72)	4.87 (2.53)
Liver function test increased	9	8.91 (4.63–17.15)	3.15 (1.40)	8.87 (62.86)	8.87 (4.61)
Burning sensation	9	4.51 (2.34–8.68)	2.17 (0.82)	4.49 (24.46)	4.49 (2.33)
Cholelithiasis	8	10.29 (5.14–20.62)	3.36 (1.37)	10.26 (66.81)	10.25 (5.12)
Tendon injury	8	98.28 (48.93–197.39)	6.60 (2.09)	97.92 (760.56)	97.05 (48.32)
Blood creatinine increased	8	4.04 (2.02–8.09)	2.01 (0.63)	4.03 (18.23)	4.03 (2.01)
Hyperuricaemia	7	54.43 (25.87–114.52)	5.75 (1.80)	54.26 (364.10)	53.99 (25.66)
Pancreatitis	6	4.61 (2.07–10.28)	2.20 (0.51)	4.60 (16.92)	4.60 (2.06)
Pancreatitis acute	6	9.19 (4.12–20.49)	3.20 (0.99)	9.17 (43.66)	9.16 (4.11)
International normalized ratio increased	6	15.86 (7.11–35.36)	3.98 (1.25)	15.82 (83.18)	15.80 (7.08)
Tendon disorder	6	32.95 (14.77–73.51)	5.03 (1.47)	32.86 (184.8)	32.76 (14.69)
Rhabdomyolysis	6	5.52 (2.48–12.31)	2.46 (0.65)	5.51 (22.14)	5.51 (2.47)
Angina pectoris	5	7.08 (2.94–17.03)	2.82 (0.63)	7.07 (26.02)	7.06 (2.94)
Musculoskeletal discomfort	5	8.49 (3.53–20.42)	3.08 (0.73)	8.47 (32.92)	8.46 (3.52)
Abnormal faeces	4	12.93 (4.84–34.50)	3.69 (0.64)	12.91 (43.89)	12.89 (4.83)
Biliary colic	4	45.14 (16.89–120.62)	5.49 (0.90)	45.06 (171.6)	44.87 (16.79)
Ligament rupture	4	28.43 (10.65–75.92)	4.82 (0.84)	28.38 (105.39)	28.31 (10.60)
Muscle rupture	4	31.15 (11.66–83.18)	4.95 (0.85)	31.09 (116.17)	31.01 (11.61)
Blood creatine phosphokinase increased	4	6.01 (2.25–16.04)	2.59 (0.29)	6.00 (16.68)	6.00 (2.25)
Glomerular filtration rate decreased	4	7.96 (2.98–21.24)	2.99 (0.44)	7.95 (24.29)	7.95 (2.98)
Muscle twitching	4	6.29 (2.36–16.77)	2.65 (0.32)	6.28 (17.74)	6.27 (2.35)
Tendon discomfort	4	135.27 (50.41–362.98)	7.06 (0.98)	135.03 (525.54)	133.36 (49.70)
Chromaturia	4	6.79 (2.54–18.10)	2.76 (0.36)	6.78 (19.69)	6.77 (2.54)
Micturition urgency	4	11.37 (4.26–30.33)	3.50 (0.59)	11.35 (37.71)	11.34 (4.25)
Cholecystitis acute	3	21.48 (6.92–66.74)	4.42 (0.36)	21.46 (58.39)	21.41 (6.89)
Ligament sprain	3	7.96 (2.56–24.70)	2.99 (0.09)	7.95 (18.20)	7.94 (2.56)
Lipids abnormal	3	127.97 (40.96–399.77)	6.98 (0.51)	127.80 (372.97)	126.30 (40.43)
Joint noise	3	25.88 (8.33–80.42)	4.69 (0.39)	25.85 (71.49)	25.79 (8.30)
Rotator cuff syndrome	3	8.14 (2.62–25.28)	3.02 (0.10)	8.13 (18.76)	8.13 (2.62)
Urine abnormality	3	17.09 (5.50–53.07)	4.09 (0.32)	17.07 (45.31)	17.04 (5.49)

ADEs = adverse drug events, BAPEFC = bempedoic acid plus ezetimibe fixed-dose combination, CI = confidence interval, EBGM = empirical Bayes geometric mean, EBGM05 = the lower limit of 95% CI, of the EBGM, IC= information component, IC025 = the lower limit of 95% CI, of the IC, PRR = proportional reporting ratio, PTs = preferred terms, ROR = reporting odds ratio.

All 4 algorithms are positive.

**Figure 5.**
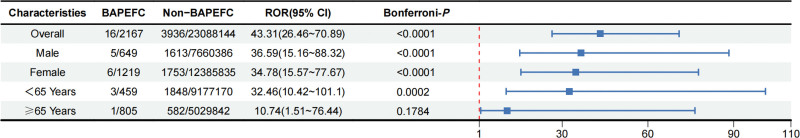
Subgroup analysis of the signal for “Blood Triglycerides Increased” by gender and age. ROR values with 95% confidence intervals are shown. Statistical significance after Bonferroni correction is indicated (Bonferroni-*P* < .05). BAPEFC = bempedoic acid plus ezetimibe fixed-dose combination, CI = confidence interval, ROR = reporting odds ratio.

**Figure 6.**
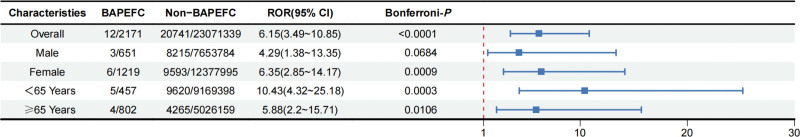
Subgroup analysis of the combined signal for “Pancreatitis” and “Pancreatitis Acute” by gender and age. ROR values with 95% confidence intervals are shown. Statistical significance after Bonferroni correction is indicated (Bonferroni-*P* < .05). BAPEFC = bempedoic acid plus ezetimibe fixed-dose combination, CI = confidence interval, ROR = reporting odds ratio.

Subgroup analyses were performed for the PTs not mentioned in the prescribing information, namely “Blood Triglycerides Increased,” “Pancreatitis,” and “Pancreatitis Acute.” For blood triglycerides increased, a positive ROR signal was observed in both the male and female subgroups (ROR = 36.59 [95% CI: 15.16–88.32] vs ROR = 34.78 [95% CI: 15.57–77.67]), and the adjusted Bonferroni-*P* values were statistically significant in both subgroups (Bonferroni-*P* < .0001). Positive ROR signals were also found in both the non-elderly (<65 years) and elderly (≥65 years) age subgroups (ROR = 32.46 [95% CI: 10.42–101.10] vs ROR = 10.74 [95% CI: 1.51–76.44]); however, the adjusted Bonferroni-*P* value was statistically significant only in the non-elderly subgroup (Bonferroni-*P* = .0002; Fig. 5). After combining the data for pancreatitis and pancreatitis acute, subgroup analysis revealed positive ROR signals in both the non-elderly and elderly age subgroups (ROR = 10.43 [95% CI: 4.32–25.18] vs ROR = 5.88 [95% CI: 2.2–15.71]), with statistically significant adjusted Bonferroni-*P* values for both (Bonferroni-*P* = .0003 vs Bonferroni-*P* = .0106). Positive ROR signals were also observed in both the male and female subgroups (ROR = 4.29 [95% CI: 1.38–13.35] vs ROR = 6.35 [95% CI: 2.85–14.17]), but the adjusted Bonferroni-*P* value was statistically significant only in the female subgroup (Bonferroni-*P* = .0009; Fig. 6).

## 4. Discussion

This study represents the first systematic signal mining and evaluation of the real-world safety profile of the BAPEFC, utilizing the US FAERS database. Through the analysis of 875 relevant adverse event reports from the first quarter of 2020 to the first quarter of 2025, we identified several adverse reactions with significant signal strengths, which not only corroborated certain known risks but also revealed potential safety signals not explicitly mentioned in the prescribing information.

A total of 47 PTs for adverse events, spanning 10 SOCs, were consistently identified as positive signals by all 4 detection methods (ROR, PRR, BCPNN, and MGPS). Among these, musculoskeletal and connective tissue disorders were the most frequently reported SOC, accounting for 24.31% of all reports, with a significantly high ROR value of 6.02 (95% CI: 5.46–6.63). Specific manifestations included common symptoms such as arthralgia, myalgia, and pain in extremity. Ballantyne et al,^[8]^ in a phase 3, multicenter, double-blind study across 78 US research centers, reported musculoskeletal adverse events in 7.1% of the BAPEFC group, compared with 8.0% in the bempedoic acid monotherapy group and 8.1% in the ezetimibe monotherapy group. Although the incidence in the combination group was lower than in the monotherapy groups in that trial, our study found the highest proportion of reports for this SOC (24.31%). Therefore, clinicians should remain vigilant for musculoskeletal symptoms during treatment with the BAPEFC.^[6,13,14]^ Furthermore, tendon-related adverse reactions showed prominent positive signals, including tendon rupture, tendon pain, tendonitis, tendinopathy, tendon injury, and tendon discomfort. The most serious among these is tendon rupture. A potential mechanism could be that bempedoic acid, by inhibiting adenosine triphosphate-citrate lyase, reduces the generation of acetyl-CoA in the tricarboxylic acid cycle, potentially affecting mitochondrial function and inducing musculoskeletal and connective tissue-related adverse reactions. Tendon cells, with their inherently low metabolic rate and weak repair capacity, might be more susceptible to energy deprivation, leading to impaired collagen synthesis, increased tendon brittleness, and a higher risk of tendon injury and rupture. A double-blind, randomized, placebo-controlled cardiovascular outcomes trial involving 13,970 patients^[15]^ found a higher incidence of tendon rupture events in the bempedoic acid group compared with the placebo group (1.2% vs 0.9%). Tendon ruptures may occur more frequently in patients over 60 years of age, those taking concomitant corticosteroids or fluoroquinolones, patients with a history of tendon disorders, renal impairment, or diabetes, most commonly involving the Achilles tendon, biceps tendon, or rotator cuff.^[16–19]^ Notably, although the number of reports for tendon discomfort was small (n = 4), it exhibited the highest signal strength (ROR = 135.27, PRR = 135.03), suggesting a strong potential association with the drug and warranting significant clinical attention.

Regarding adverse events related to the renal and metabolic systems, we identified several signals that were positive across all 4 algorithms, including blood uric acid increased, renal impairment, micturition urgency, blood creatinine increased, glomerular filtration rate decreased, gout, and hyperuricaemia. Ballantyne et al^[8]^ found that the incidence of increased blood uric acid and increased blood creatinine was higher in the BAPEFC group (3.5%) compared with the bempedoic acid monotherapy group (1.1%) and the ezetimibe monotherapy group (0%). The incidence of urinary tract infections was also higher in the combination group (5.9% vs 3.4% vs 2.3%), which aligns with the positive signal for micturition urgency found in our study. Nissen et al^[15]^ reported higher incidences of increased blood uric acid (10.9% vs 5.6%), gout (3.1% vs 2.1%), and renal impairment (11.5% vs 8.6%) in the bempedoic acid group compared with the placebo group. Decreased glomerular filtration rate and increased serum creatinine have been observed in clinical trials and post-marketing surveillance. The exact mechanism remains incompletely understood but is thought to involve bempedoic acid acting as a weak inhibitor of organic anion transporter 2, potentially affecting the transport and excretion of uric acid and the renal tubular secretion of creatinine, leading to mild elevations in plasma uric acid and creatinine.^[20–22]^ If symptoms of hyperuricaemia occur, serum uric acid levels should be monitored, and urate-lowering therapy should be initiated when clinically indicated to prevent progression to gout. Our findings further validate this risk in a real-world setting, suggesting the need for monitoring uric acid levels during long-term use, especially in high-risk populations.

Within the hepatobiliary and digestive systems, adverse reactions yielding positive signals across all 4 algorithms included hepatic enzyme increased, cholelithiasis, biliary colic, and cholecystitis acute. Ballantyne et al^[8]^ reported a higher incidence of elevated hepatic enzymes in the BAPEFC group compared with the placebo group (1.2% vs 0%). Similarly, Laufs et al^[23]^ found a higher incidence of elevated liver enzymes in the bempedoic acid group versus the placebo group (16% vs 2%). Hepatic enzyme elevation is considered potentially related to the intrahepatic activation of bempedoic acid and subsequent hepatocyte stress induced by the drug or its metabolites. Nissen et al^[15]^ reported a higher incidence of cholelithiasis in the bempedoic acid group compared with the placebo group (2.2% vs 1.2%). Biliary colic and acute cholecystitis are closely associated with gallstone formation. The mechanism behind cholelithiasis with BAPEFC is currently unclear but may involve alterations in lipid metabolism that change bile composition, thereby increasing the risk of gallstone formation; the specific mechanism requires further investigation.^[24]^ The incidence of cholelithiasis may be increased in high-risk patients. Therefore, it is important to identify high-risk individuals and educate patients to recognize symptoms. Routine gallbladder ultrasound screening is not necessary, but persistent right upper quadrant pain should prompt timely investigation.

Angina pectoris emerged as a newly identified adverse reaction signal, which is inconsistent with some existing research suggesting that lipid-lowering drugs can improve cardiac metabolism, reduce myocardial ischemia, and thus alleviate angina symptoms. However, Han et al,^[6]^ in a study on ezetimibe adverse reactions, identified unstable angina as a newly discovered strong signal (n = 120, ROR = 30.53, PRR = 30.41, IC = 4.9, EBGM = 29.85). They hypothesized that ezetimibe-induced depression leading to elevated blood pressure could be a potential cause for unstable angina in patients. Conversely, Ballantyne et al^[8]^ found that the incidence of acute myocardial infarction was lower in the BAPEFC group (1.2%) compared with the bempedoic acid monotherapy (2.3%) and ezetimibe monotherapy (3.5%) groups, further supporting the role of the combination’s dual mechanism and synergistic lipid-lowering effect in reducing adverse cardiovascular events like acute myocardial infarction.^[25]^ Crucially, patients prescribed the BAPEFC often have underlying severe cardiovascular disease. Any drug effect or natural progression of the underlying disease could be a trigger. If patients experience symptoms suggestive of angina, such as chest pain, chest tightness, or pressure, they should seek prompt medical attention for evaluation and potential medication adjustment.

This study also identified several adverse event signals not explicitly listed in the drug’s prescribing information, including blood triglycerides increased, pancreatitis, and pancreatitis acute. These signals were positive across all 4 algorithms and showed certain sex and age differences in subgroup analyses. Blood triglycerides increased showed a notable positive signal (n = 16, ROR = 42.83, PRR = 42.52, IC = 5.4, EBGM = 42.36). Wang et al^[5]^ also found a significant positive signal for increased triglycerides in a study of bempedoic acid adverse events (n = 24, ROR = 47.93, PRR = 46.13, IC = 5.97, EBGM = 46.19). The mechanism by which the BAPEFC increases serum triglycerides is not fully elucidated. However, based on the pharmacological actions of its components, it may be related to bempedoic acid’s interference with the complex lipid metabolism network. Additionally, the blockade of the Niemann-Pick C1-like 1 protein by ezetimibe might lead to compensatory increased dietary fat absorption in the intestine, providing more substrates for triglycerides synthesis. Blood triglycerides increased was a significant signal in both males and females, and stronger in the non-elderly subgroup (<65 years). This finding aligns with trends of mild triglycerides elevation noted in some studies on bempedoic acid, though its clinical significance requires further investigation. The combined analysis of pancreatitis and pancreatitis acute showed more significant signals in the non-elderly and female subgroups. Although the number of reports was limited, their ROR values were all >4, suggesting a potential association. Given previous case reports of pancreatitis with ezetimibe-statin combinations, it is worth exploring whether BAPEFC indirectly increases pancreatitis risk by affecting lipid metabolism or bile composition.

This study employed 4 different signal detection methods (ROR, PRR, BCPNN, and MGPS), leveraging their respective advantages to ensure sensitivity while enhancing specificity through multiple verification steps. In particular, the BCPNN and MGPS methods, incorporating Bayesian shrinkage, effectively reduced the risk of false-positive signals from small sample sizes, enhancing the reliability of the results. However, this study has several limitations. First, the FAERS is a spontaneous reporting system subject to reporting biases, under-reporting, duplicate reporting, and incomplete information. Second, the lack of age information in 54.1% of reports limited our ability to conduct a deeper analysis of risk stratification by age. Furthermore, FAERS data does not allow for the calculation of incidence rates and cannot directly infer causality, only suggesting potential associations. Finally, as BAPEFC was only marketed in 2020, long-term safety data remain limited, necessitating further validation through subsequent studies with larger sample sizes and longer follow-up durations.

## 5. Conclusion

This study conducted a comprehensive safety signal-mining analysis of the bempedoic acid and ezetimibe fixed-dose combination based on the FAERS database. It confirmed known risks associated with the musculoskeletal and metabolic systems and, for the first time in a real-world setting, identified potential new signals, such as increased blood triglycerides and pancreatitis. These findings provide crucial reference information for clinicians and suggest enhanced monitoring for the aforementioned adverse reactions when prescribing this drug. Future prospective studies, pharmacoepidemiological investigations, and mechanistic research are warranted to further validate these signals and elucidate their biological mechanisms, thereby providing more robust evidence for the safe clinical application of BAPEFC.

## Author contributions

**Conceptualization:** Bing Zhu, Yitong Ma, Zhenyan Fu.

**Methodology:** Bing Zhu, Jun Cui.

**Data curation:** Bing Zhu, Qiqi Shao.

**Visualization:** Bing Zhu.

**Supervision:** Yitong Ma, Qiqi Shao.

**Validation:** Qiqi Shao, Jun Cui.

**Writing – original draft:** Bing Zhu.

**Writing – review & editing:** Bing Zhu, Yitong Ma, Qiqi Shao, Zhenyan Fu.
